# The Wastewater Resistome: A Shotgun Metagenomics Analysis of Urban Treatment Plants in Sicily

**DOI:** 10.3390/antibiotics15020148

**Published:** 2026-02-02

**Authors:** Roberta Magnano San Lio, Andrea Maugeri, Martina Barchitta, Giuliana Favara, Maria Clara La Rosa, Claudia La Mastra, Margherita Ferrante, Antonella Agodi

**Affiliations:** Department of Medical and Surgical Sciences and Advanced Technologies “GF Ingrassia”, University of Catania, 95123 Catania, Italy; robertamagnanosanlio@unict.it (R.M.S.L.); andrea.maugeri@unict.it (A.M.); martina.barchitta@unict.it (M.B.); giuliana.favara@unict.it (G.F.); mariaclara.larosa@unict.it (M.C.L.R.); claudia.lamastra@unict.it (C.L.M.); marfer@unict.it (M.F.)

**Keywords:** wastewater treatment plants, antimicrobial resistance, metagenome

## Abstract

**Background/Objectives:** Antimicrobial resistance (AMR) in wastewater represents a valuable reservoir of information for wastewater-based epidemiology (WBE) and a major environmental and public health concern, as wastewater treatment plants (WWTPs) are recognized hotspots for the accumulation and dissemination of antimicrobial resistance genes (ARGs). Within the One Health framework, and to better understand the contribution to AMR spread and the potential of metagenomic surveillance, this study aimed to characterize the taxonomic, functional, and resistome profiles of three WWTPs in Sicily, specifically those located in Catania, Giarre, and Syracuse. **Methods:** Sixty-nine composite influent samples were collected between February 2022 and December 2023. Shotgun metagenomic sequencing was performed on the Illumina NovaSeq platform. Bioinformatic analyses were conducted to assess microbial community composition, functional pathways, and ARG prevalence across sites. **Results:** Dominant genera included *Aliarcobacter*, *Bacteroides*, and *Acinetobacter*. Site-specific taxonomic variations reflected differences in local microbial ecology. Functional profiling revealed enrichment in membrane-associated, ribosomal, and energy metabolism pathways, consistent with the expected functional redundancy of wastewater microbiomes. Resistome analysis detected a diverse and ubiquitous array of ARGs, dominated by β-lactam and macrolide resistance genes, followed by aminoglycoside, sulphonamide, and tetracycline classes. **Conclusions:** These findings highlight urban wastewater as a relevant reservoir and dissemination route for AMR and support the integration of metagenomic approaches into wastewater surveillance programs. By providing region-specific, integrated taxonomic, functional, and resistome data from Sicilian WWTPs, this study contributes to the growing body of evidence supporting WBE as a valuable tool for AMR monitoring and One Health-oriented risk assessment.

## 1. Introduction

Antimicrobial resistance (AMR) is increasingly recognized as one of the most critical public health challenges of the 21st century [1]. Addressing water as a key environmental driver of AMR is essential, given its central role in sustaining ecological, human, and economic systems. In this scenario, wastewater represents a favorable setting for the proliferation of antimicrobial-resistant bacteria and serves as a valuable matrix for monitoring antibiotics, microbial communities, and antimicrobial resistance genes (ARGs) [2]. Identifying hotspots of ARG dissemination starts by determining their occurrence in urban wastewater treatment plants (WWTPs), which receive wastewater from diverse sources such as hospitals and households [3].

The presence of shared and persistent ARGs—referred to as the core resistome—has been reported, typically including resistance determinants against widely used antibiotic classes such as tetracyclines, β-lactams, and glycopeptides [4]. Regardless of local selective pressures, the existence of such a core set of ARGs suggests that these resistance traits are stably maintained across environments and microbial communities, increasing the likelihood of their dissemination through horizontal gene transfer (HGT) [4,5,6,7].

For these reasons, metagenomic techniques for the analysis of ARGs are essential to monitor these environments and to develop tailored surveillance strategies aimed at combating the spread of AMR, decreasing the risk that clinically important pathogens may acquire resistance and undermine the effectiveness of critical antibiotic therapies. Despite several limitations—such as the lack of long-term longitudinal studies, difficulty in identifying true ARG reservoirs, and challenge of detecting rare but clinically relevant genes—short-read next-generation sequencing enables the simultaneous quantification of numerous ARGs within a single sample, while also providing information on bacterial species, pathogens, and virulence factors [8]. Beyond the resistome, the combined taxonomic and functional profiling of wastewater microbiomes allows for a comprehensive assessment of microbial diversity, community dynamics, and metabolic pathways potentially linked to ARG maintenance and dissemination [9].

While wastewater resistome studies are increasingly reported worldwide, Southern European regions, and particularly Southern Italy, remain poorly characterized using shotgun metagenomic approaches applied to urban wastewater influents [3]. As a result, region-specific baseline data integrating taxonomic, functional, and resistome profiles from municipal wastewater are still scarce. As part of the PNRR Sicilian Micro and Nano Technology Research and Innovation Center—SAMOTHRACE project, the present study analyses 69 monthly composite DNA samples collected from three WWTPs in Sicily using shotgun metagenomic sequencing, with the aim of characterizing their taxonomic, functional, and antibiotic-resistance profiles.

## 2. Results

### 2.1. Sequencing Summary and Quality Control

Sequencing performance was consistently high across all samples, indicating robust library preparation and run reproducibility (Figure S1). Per-sample raw sequencing output averaged approximately 18.5 million paired-end read pairs (2 × 150 bp), with limited between-sample variability. After adapter trimming and quality filtering, high-quality reads constituted the majority of the dataset, with HQ Reads routinely exceeding 97%. Base-calling quality was similarly stable, as reflected by HQ Bases (Q30) values around 97–98%, indicating that most bases achieved Phred scores ≥ 30. GC content remained tightly distributed (≈41–42%), in line with expectations for this sample type, and mean read length (≈148 bp) was uniform across the cohort, suggesting minimal library degradation or batch effects. Host-screening and read classification further indicated low levels of host contamination. Across samples, host-classified reads typically accounted for ~0.2–2.7% of total reads, leaving ~97.3–99.8% non-host reads for microbial and environmental profiling. On average, the proportion of taxonomically classified reads was ~25.3% per sample with approximately 4.5 million paired-end read pairs per sample.

### 2.2. Taxonomic Composition

**Figure 1:**
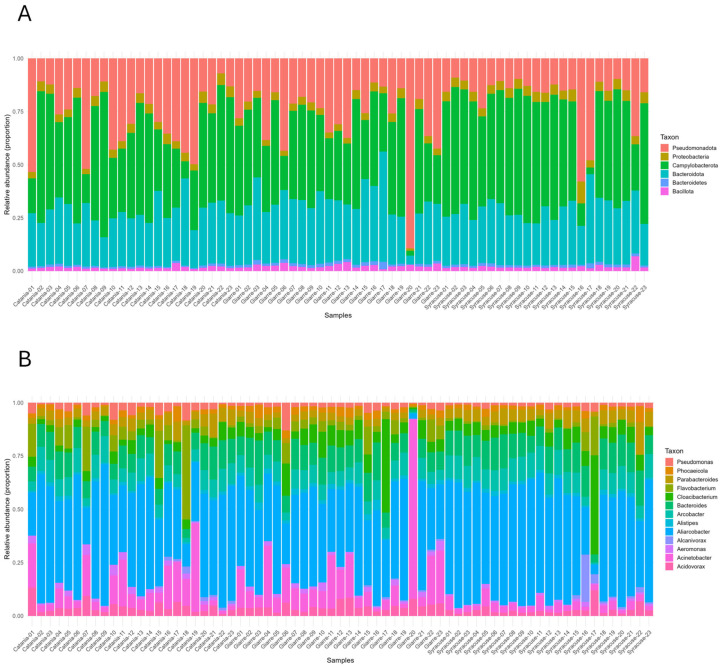

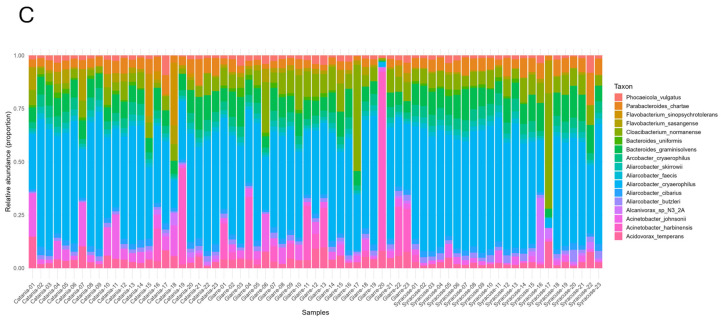
Community composition at the phylum, genus, and species level. Relative abundances at the phylum (**A**), genus (**B**), and species (**C**) level across 69 environmental metagenomes (Catania, Giarre, Syracuse; n = 23 per site). Bars are ordered by site and sample ID.

Taxonomic classification revealed a broadly consistent structure across the 69 metagenomes from Catania, Giarre, and Syracuse, while still capturing site-level differences in relative abundance (Figure 1). At the phylum level, communities were dominated by *Campylobacterota*, *Bacteroidota*, and *Pseudomonadota*, which together accounted on average for 41.5%, 26.8%, and 24.4% of signal across the cohort, respectively; smaller contributions arose from *Proteobacteria* (4.1%), *Bacillota* (2.0%), and Bacteroidetes (1.2%). Site-level means preserved this overall architecture but showed modest re-weighting: Syracuse exhibited a higher *Campylobacterota* fraction (47.7%) and lower *Pseudomonadota* (18.1%), Giarre displayed a more balanced split between *Bacteroidota* and *Pseudomonadota* (28.8% and 27.9%), and Catania lay between these extremes (*Campylobacterota* 40.6%, *Pseudomonadota* 27.3%, *Bacteroidota* 25.4%) (Figure 1A). At genus level, *Aliarcobacter* was predominant (42.5%), followed by *Bacteroides* (11.0%), *Acinetobacter* (9.5%), *Arcobacter* (8.3%), *Cloacibacterium* (6.6%), and *Flavobacterium* (4.7%) (Figure 1B). Species-level profiles were dominated by *Aliarcobacter cryaerophilus* (44.5%), with additional contributions from *Bacteroides graminisolvens* (9.0%), *Cloacibacterium normanense* (8.5%), *Parabacteroides chartae* (5.1%), *Acinetobacter harbinensis* (4.6%), and *Arcobacter cryaerophilus* (4.3%) (Figure 1C). These results delineate a shared taxonomic backbone across locations and a tendency toward a more mono-dominant configuration in Syracuse, consistent with its higher *Campylobacterota* burden.

**Figure 2:**
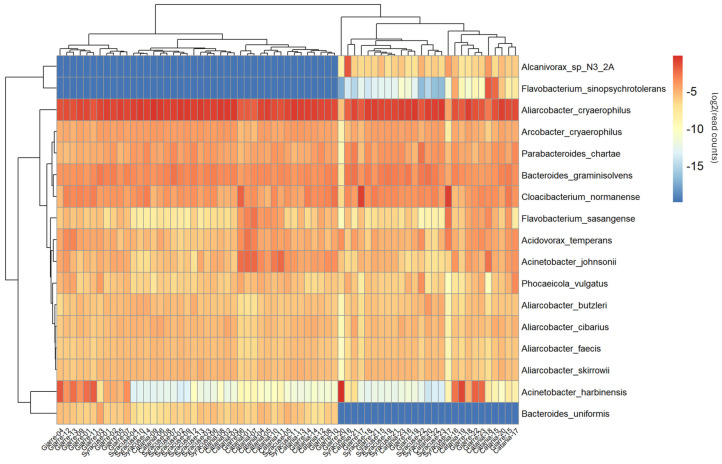
A species-level heatmap with hierarchical clustering. A heatmap showing the taxonomic abundance and their relation across the samples. Dendrograms determined by computing hierarchical clustering from the abundance levels shows the relationship between the species and the samples. The abundance levels (number of reads associated with each taxa) are logarithmically transformed to base 2 for clarity.

At the species level, the taxonomic heatmap based on log_2_-transformed relative abundances supported these trends and revealed a clear yet incomplete site effect (Figure 2).

Samples from Catania and Giarre tended to cluster in adjacent clades, and Syracuse formed a partially compact group, although several samples from all three locations were interspersed across branches. This partial separation indicates that between-site differences coexist with within-site heterogeneity. The clustering structure was driven primarily by gradients in *Campylobacterota*—particularly *Aliarcobacter*/*Arcobacter*—counterbalanced by Bacteroidota (e.g., *Bacteroides*, *Cloacibacterium*) and *Pseudomonadota* (e.g., *Acinetobacter*).

**Figure 3:**
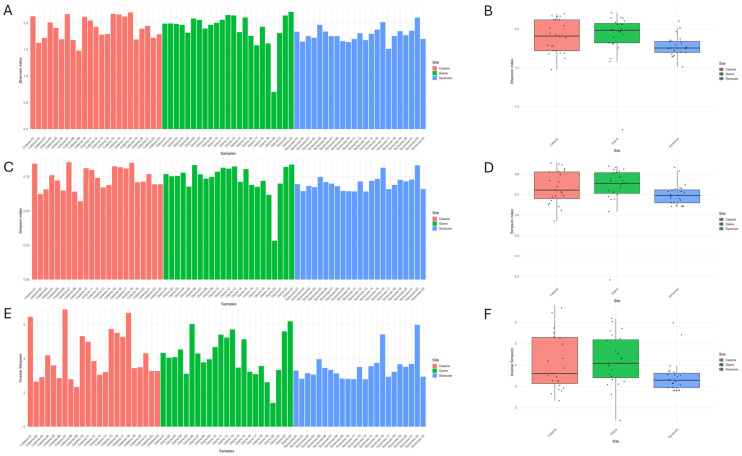
Diversity indices computed based on the species counts found in each sample. (**A**,**B**) Shannon diversity at the species level for each sample and by site. (**C**,**D**) Simpson diversity at the species level for each sample and by site. (**E**,**F**) Inverse Simpson diversity at the species level for each sample and by site.

Alpha diversity summarized as the median (IQR) was comparable in Catania and Giarre and reduced in Syracuse: Shannon was 1.910 (1.720–2.120), 1.984 (1.822–2.074), and 1.756 (1.698–1.842) for Catania, Giarre, and Syracuse, respectively (Figure 3A,B); Simpson was 0.722 (0.680–0.811), 0.755 (0.706–0.807), and 0.696 (0.660–0.724) (Figure 3C,D); and inverse Simpson was 3.600 (3.130–5.299), 4.087 (3.402–5.191), and 3.290 (2.939–3.619) (Figure 3E,F). Taken together, these concordant trends point to greater dominance and reduced diversity in Syracuse, consistent with the compositional profiles.

### 2.3. Gene Ontology Annotations

The functional potential inferred from Gene Ontology (GO) annotations indicated a conserved core of cellular and metabolic functions across all sampling sites, consistent with the well-documented functional redundancy of wastewater microbiomes. Highly abundant GO terms were dominated by housekeeping and structural categories, including membrane-associated components (e.g., integral component of membrane, GO:0016021), ribosomal structure, and nucleic acid-binding activities. These functions showed only modest variation in relative abundance among sites, reflecting strong selective pressure for essential metabolic and translational processes in engineered wastewater environments. Taxonomic stratification of GO:0016021 linked this conserved functional signal to dominant bacterial taxa—such as *Aliarcobacter cryaerophilus*, *Bacteroides graminisolvens*, *Cloacibacterium normanense*, and *Acinetobacter* spp.—indicating that similar functional roles are maintained by different community members across locations. While GO-based profiling primarily captures broad, conserved biological functions, subtle site-specific differences in taxonomic contributors suggest that functional stability is achieved through compositional variability. Overall, the annotations support metabolically active, structurally robust communities in which membrane processes, energy metabolism, and translation represent core and ubiquitous capabilities of wastewater microbiomes.

### 2.4. Resistome Profiles

**Figure 4:**
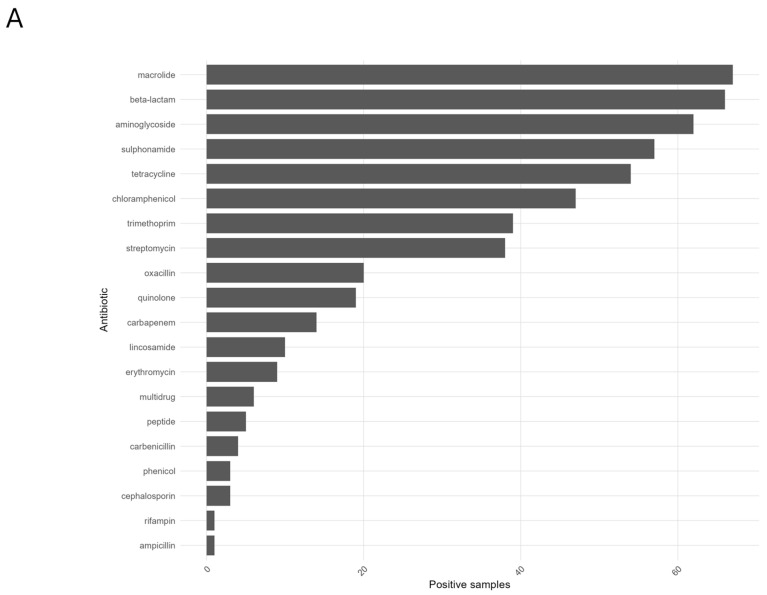

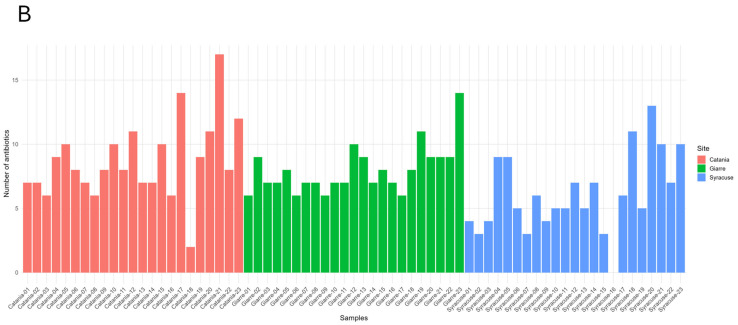
ARG profiling metrics. (**A**) Top 20 most prevalent antibiotic classes ranked by number of positive samples in cohort. Bars show absolute prevalence (samples positive). (**B**) Number of antibiotic classes detected per sample.

The resistome was broad and ubiquitous at the class level. The most prevalent classes were macrolides (67/69, 97.1%), β-lactams (66/69, 95.7%), aminoglycosides (62/69, 89.9%), sulphonamides (57/69, 82.6%), and tetracyclines (54/69, 78.3%), followed by chloramphenicol (47/69, 68.1%), trimethoprim (39/69, 56.5%), streptomycin (38/69, 55.1%), oxacillin (20/69, 29.0%), and quinolones (19/69, 27.5%) (Figure 4A).

**Figure 5:**
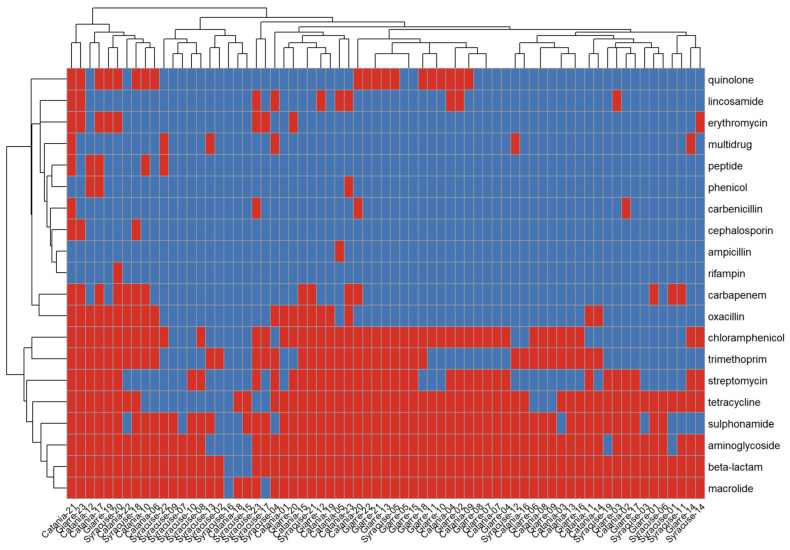
A heatmap of antibiotic class presence. A heatmap showing the antibiotic class presence (in red versus blue) and their relation across the samples. Dendrograms determined by computing hierarchical clustering from the abundance levels shows the relationship between the species and the samples.

Site-level prevalences were consistently high in Catania and Giarre and lower in Syracuse; for example, macrolides were detected in 100% of samples in Catania and Giarre versus 91.3% in Syracuse, a pattern mirrored by β-lactams (95.7% and 100% vs. 91.3%), aminoglycosides (95.7% and 100% vs. 73.9%), tetracyclines (87.0% and 91.3% vs. 56.5%), and chloramphenicol (78.3% and 87.0% vs. 39.1%) (Figure 4B). At the sample level, the number of ARG classes per sample averaged 7.6 ± 2.9 overall (median 7; range 0–17), with broader repertoires in Catania (8.7 ± 3.1, range 2–17) and Giarre (8.0 ± 1.9, range 6–14) and a narrower profile in Syracuse (6.1 ± 3.1, range 0–13). The ARG heatmap displayed extensive cohort-wide “blocks” for macrolides, β-lactams, and aminoglycosides; Catania and Giarre more frequently co-clustered, whereas Syracuse showed lower intensity across several classes (Figure 5). As with taxonomy, the site signal in the resistome was evident but not absolute, consistent with a shared multi-class resistome modulated by location-specific differences in class prevalence.

## 3. Discussion

The metagenomic analysis of wastewater samples from three Sicilian WWTPs provides valuable insights into the structure, functionality, and resistome of microbial communities. Although the three sites—Catania, Giarre, and Syracuse—shared a broadly similar core microbiome dominated by bacteria, distinct site-specific patterns emerged in both taxonomic and functional profiles, reflecting differences in environmental pressures, urban inputs, and ecological stability [8]. From an ecological perspective, the variability observed among the three sampling sites likely reflects differences in local environmental conditions, anthropogenic load, and microhabitat composition influencing microbial community structure [10,11]. Samples from Syracuse exhibited a more homogeneous and mono-dominant microbial composition, suggesting the predominance of well-characterized taxa such as *Aliarcobacter cryaerophilus*, typically associated with wastewater environments characterized by lower taxonomic variability and higher community dominance. In contrast, Catania samples displayed a more heterogeneous community composition, with a higher fraction of unclassified or low-abundance taxa, potentially reflecting adaptation to local stressors, such as industrial discharges or variable urban inputs. Giarre, on the other hand, showed a balanced microbial structure with intermediate diversity indices, consistent with mixed influences from agricultural runoff and urban effluents [12,13]. Across all sites, *Aliarcobacter*, *Bacteroides*, *Acinetobacter*, and *Cloacibacterium* were among the most abundant genera. These taxa are frequently detected in wastewater environments and include both environmental and opportunistic species capable of biofilm formation and HGT [3,14,15]. Their persistence across all samples supports the existence of a core microbiome resilient to environmental fluctuations and anthropogenic pressures [16]. The calculated diversity indices further confirmed moderate-to-high ecological evenness, suggesting balanced community interactions and limited dominance by single species [17].

Functional profiling revealed enrichment in membrane-associated processes (GO:0016021), ribosomal structure, and energy metabolism pathways across sites. These functions are typical of metabolically active and structurally resilient microbial communities, reflecting the ecological necessity of maintaining cell integrity, nutrient transport, and protein synthesis under variable physicochemical conditions [18,19,20]. The high presence of membrane transport genes, particularly in Giarre samples, may reflect an increased representation of membrane-associated functions, potentially linked to nutrient exchange processes in chemically complex environments, potentially influenced by agricultural or runoff inputs [21,22]. Syracuse samples displayed the highest functional homogeneity, consistent with the dominance of *Aliarcobacter* and enrichment in categories related to ATP binding, metal ion metabolism, and ribosomal function—features consistent with microbial communities exhibiting high functional homogeneity and dominance of core metabolic processes [9]. Conversely, Catania samples showed slightly lower functional evenness and a reduced contribution of energy-related processes, suggesting increased temporal or compositional variability potentially linked to fluctuating environmental inputs [17,23]. Collectively, these findings underline the existence of a functionally redundant microbial core that supports community resilience despite environmental heterogeneity.

Resistome profiling highlighted the ubiquitous presence of ARGs associated with macrolides and β-lactams across all samples, followed by genes conferring resistance to aminoglycosides, sulphonamides, and tetracyclines. These ARG classes are among the most commonly detected in wastewater and mirror the global pattern of resistance observed in urban and hospital effluents. Their widespread distribution supports the hypothesis that wastewater serves as a critical hotspot for the maintenance and dissemination of multidrug resistance determinants [8,24]. Catania and Giarre displayed the highest cumulative ARG abundance, followed by Syracuse. The lower ARG load in Syracuse aligns with its reduced taxonomic diversity and mono-dominant microbial profile, which may limit horizontal gene transfer potential. In contrast, the more heterogeneous ARG distribution in Catania may reflect variable urban discharges or fluctuating microbial communities.

The consistent detection of β-lactam and macrolide resistance genes across all sites emphasizes their role as part of a shared core resistome, likely maintained through stable horizontal gene transfer mechanisms within diverse bacterial hosts. The co-occurrence of taxa such as *Acinetobacter* and *Aliarcobacter*—both detected in high abundance and known reservoirs of mobile genetic elements (MGEs)—further supports a strong potential for ARG dissemination through HGT. This interpretation aligns with previous studies demonstrating that wastewater ecosystems act as reservoirs for ARG–MGE linkage, promoting the persistence and spread of clinically relevant resistance genes in downstream environments [25,26,27]. The observed ARG profiles are broadly consistent with earlier reports from European and Mediterranean WWTPs, where β-lactam, macrolide, and aminoglycoside resistance genes were dominant [28,29]. However, the detection of site-specific differences in ARG abundance and composition underscores the influence of local environmental and infrastructural factors, including antibiotic consumption patterns, wastewater treatment efficiency, and industrial contributions [3].

This work has several limitations that should be acknowledged. First, the study was designed as a site-resolved characterization of three urban WWTPs rather than a Sicily-wide survey. As such, the findings provide localized evidence that should not be extrapolated to all WWTPs in the region. Moreover, physicochemical and operational metadata (e.g., pH, conductivity, temperature, nutrient loads, flow rates) were not available for the analyzed samples, limiting our ability to relate microbial composition and ARG patterns to environmental or process covariates. Future studies including a broader set of WWTPs and collecting standardized metadata will strengthen regional inference.

Second, short-read shotgun metagenomics limits the reconstruction of genomic context and therefore constrains resolution of ARG–mobile genetic element (MGE) associations (e.g., plasmid versus chromosomal localization, co-localization with integrons/IS/ICE). We did not perform a dedicated MGE analysis; this was beyond the scope of the present work and will be prioritized in follow-up studies using assembly-based pipelines and targeted MGE annotation. In addition, low-abundance but clinically relevant ARGs may fall below detection thresholds. Complementary targeted qPCR/dPCR and/or long-read/Hi-C approaches would improve sensitivity and resolve genomic linkage. As with all reference-based methods, taxonomy and functional assignments are conditioned by database coverage and curation, which can be limiting in environmental metagenomes and contribute to the fraction of unclassified reads.

Third, taxonomic profiles were expressed as Total Sum Scaling (TSS) proportions after host-read removal. TSS is appropriate for exploratory, comparative composition but remains compositional. It does not provide absolute loads and can be influenced by changes in other community members. We did not perform absolute normalization to 16S rRNA gene equivalents or bacterial cell counts due to the absence of those measurements. Future campaigns integrating qPCR-16S or single-copy marker-based genome equivalents (and, where feasible, spike-in standards) will enable absolute scaling alongside the present framework. In line with the analysis specification provided with the dataset, species rarefaction was computed from the post-processed species abundance table. This yields a conservative assessment of depth sufficiency for dominant taxa rather than the entire long-tail of rare species detectable in unfiltered classifications. Finally, although monthly composite DNA samples were analyzed across the study period, the design does not provide a high-frequency or multi-year time series, which limits the assessment of seasonal patterns, treatment-dependent fluctuations, and inter-annual variability in ARG dynamics and community structure. Longer time series and coordinated longitudinal sampling would allow for more robust temporal inference.

Despite certain limitations, this study is, to the best of our knowledge, the first to provide a comprehensive metagenomic characterization of urban wastewater influents from municipal WWTPs in Sicily, one of the Italian regions most affected by antimicrobial resistance. More broadly, Italy is among the European countries with a high AMR burden. Prior research in the Italian context has investigated related contexts using distinct methodologies and sample types: Ferraro et al. employed qPCR and digital PCR to quantify specific resistance genes in wastewater [30]; Curran et al. focused on sediments from the Venetian Lagoon and drainage canals, rather than municipal effluents [31]; while Gigliucci et al. applied shotgun metagenomics to biosolids and reclaimed water, not to influent or effluent from WWTPs [32]. In contrast, our study offers a broader taxonomic and functional overview of microbial communities and associated resistomes in urban wastewater streams, contributing novel data within a One Health perspective.

The consistent presence of a core resistome—dominated by β-lactam and macrolide resistance genes—across geographically distinct WWTPs underscores the relevance of integrating metagenomic surveillance into wastewater monitoring programs. Such integration could support early detection of emerging ARGs and serves as an indicator of both treatment performance and potential public health risks. Future research should prioritize complementary approaches, including targeted metagenomics, qPCR validation of clinically significant resistance genes, and metatranscriptomics to assess gene expression and activity. Simultaneously, the development of rapid, sensitive biosensors for in situ detection of key ARGs and biomarkers is essential to enable real-time monitoring and more effective AMR risk assessment. Moreover, coupling resistome profiles with contextual metadata—such as antibiotic consumption patterns, industrial discharge inputs, and treatment efficiency—would significantly enhance the predictive power of surveillance systems and inform mitigation strategies.

## 4. Materials and Methods

### 4.1. Sampling

Between February 2022 and December 2023, influent wastewater was sampled on a weekly basis at three WWTPs in Sicily—Pantano D’Arci (Catania), Syracuse, and Giarre (Catania). A summary of sampling design, DNA pooling strategy, and number of sequenced composite samples per WWTP is provided in Table 1. Samples were collected upstream of any treatment process and scheduled at the beginning of each week to ensure consistency and feasibility of sampling operations. Each 100 mL sample was collected in a polyethylene bottle with a secure cap, clearly identified with sampling date and location, and transported under refrigerated conditions (5 ± 3 °C) to preserve nucleic acid integrity. Upon arrival at the laboratory, samples were split into two 50 mL aliquots: one was immediately frozen for future confirmatory analyses, while the other was processed for ARG analysis. As described in our previous study [33], wastewater concentration was performed following a protocol adapted from [34], designed to isolate microbial components. Briefly, 45 mL of each sample was subjected to a pre-treatment step in a 56 °C water bath to reduce microbial activity, followed by rapid cooling and centrifugation at +4 °C to remove particulates. Subsequent centrifugation steps, combined with the addition of polyethylene glycol and sodium chloride, promoted microbial cell precipitation. After two hours of centrifugation, a visible pellet was obtained and used for nucleic acid extraction. The resulting pellet, containing enriched microbial DNA, was used directly for nucleic acid extraction and provided high-quality DNA suitable for downstream molecular analyses. Each weekly influent sample was processed individually up to the nucleic acid extraction step; no compositing was performed at the influent or concentration stage.

### 4.2. DNA Extraction and Sequencing

Nucleic acids were extracted using the eGENE-UP platform (bioMérieux, Marcy-l’Étoile, France) [33]. This system relies on magnetic silica particles and guanidine thiocyanate lysis to disrupt microbial cells, inactivate nucleases, and preserve nucleic acids. After binding, successive washing steps removed impurities, and elution in TE buffer (pH 8.0) provided DNA of suitable quality. To minimize short-term variability in wastewater composition, monthly composite DNA samples (i.e., the analytical units subjected to sequencing) were generated by pooling equal amounts of DNA extracted from four consecutive weekly influent samples collected at each WWTP [35]. Over the February 2022–December 2023 period, this strategy resulted in 23 composite DNA samples per site, for a total of 69 sequenced samples across the three WWTPs, providing a more representative and stable profile of ARG dynamics over time.

The monthly composite DNA samples obtained were prepared and analyzed through shotgun metagenomic sequencing at Eurofins Genomics (Anzinger Str. 7a, 85560 Ebersberg, Germany) to assess the taxonomic, functional, and antibiotic-resistance profiles.

### 4.3. Pre-Processing and Quality Control

Libraries were prepared following standard paired-end protocols and sequenced on the Illumina NovaSeq platform. Each package yielded approximately 3 Gb of raw data (±3%) and included FASTQ files containing both sequences and quality scores. Raw reads were processed with fastp [36] for quality control, which involved trimming adapters, removing low-quality bases (<Q20) using a sliding window approach, and discarding reads shorter than 30 bp. For paired-end data, only read pairs meeting quality criteria were retained. Quality metrics such as Q30 percentage and GC content were then used to evaluate sequencing and sample quality. Subsequently, host-derived reads were filtered out, and the resulting high-quality non-host reads were used for downstream analyses, including antibacterial resistance profiling (ARG detection and characterization), taxonomic profiling of microbial communities, and functional profiling of gene pathways and metabolic functions.

### 4.4. Taxonomic Profiling

Taxonomic profiling was performed with MetaPhlAn [37], a computational tool that characterizes microbial community composition (Bacteria, Archaea, Eukaryotes, and Viruses) from shotgun metagenomic data with species-level resolution. MetaPhlAn uses unique clade-specific marker genes derived from approximately 17,000 reference genomes (~13,500 bacterial and archaeal, ~3500 viral, and ~110 eukaryotic). Shotgun reads were screened against the human genome (GRCh38) using Kraken/KrakenUniq, and reads classified as human were removed with SeqKit. Reads that remained unclassified were further analyzed [38]. Kraken classifies reads by splitting them into overlapping k-mers and mapping each k-mer to the lowest common ancestor of the genomes containing it in a precomputed database [39]. For each read, a classification tree is constructed by retaining only taxa associated with its k-mers. Nodes are weighted by the number of mapped k-mers, and the path with the highest cumulative weight is used for taxonomic assignment. KrakenUniq extends this method by counting unique k-mers per taxon, reducing false positives. Species rarefaction curves were computed from the species abundance count produced by the analysis pipeline (post-decontamination, annotated/retained species). Moreover, diversity indices quantify both the richness (number of species) and evenness (distribution among species) within samples. In this study, Shannon, Simpson and inverse Simpson indices were calculated using the vegan R package (version 2.7.2) to assess species diversity, with higher values generally indicating greater ecological diversity and balance among species [40].

### 4.5. Functional Profiling

Functional profiling was conducted with HUMAnN (the HMP Unified Metabolic Analysis Network), which efficiently reconstructs the abundance of microbial pathways and molecular functions from metagenomic or metatranscriptomic sequencing data. Although originally developed within the Human Microbiome Project, HUMAnN is applicable to any microbial community [41].

### 4.6. Resistome Profiling

Resistome profiling, aimed at characterizing the diversity and dynamics of ARGs, was carried out using Graphing Resistance Out Of meTagenomes (GROOT) [42]. GROOT integrates a variation graph representation of gene sets with a locality-sensitive hashing forest indexing scheme, enabling rapid classification of metagenomic reads through similarity search. Hierarchical local alignment of classified reads against graph traversals then allows for accurate reconstruction of full-length gene sequences using a dedicated scoring system. Gene-level presence was called only if all the following were satisfied: (i) ≥5 non-redundant supporting reads; (ii) breadth of coverage ≥ 70% of the gene length (or ≥120 bp for targets < 200 bp); and (iii) mean alignment identity ≥ 90% across aligned segments. Reads that multi-map were excluded from evidence; unresolved alternative graph paths were flagged ambiguous and not counted. Class-level prevalence was defined as presence/absence per sample, with a class considered present if ≥1 constituent gene met the gene-level criteria.

### 4.7. Statistical Analyses

Taxonomic abundances were normalized by Total Sum Scaling (TSS). Specifically, after host-read removal, the reads assigned to each taxon within a sample were divided by the total number of assigned reads in that same sample, yielding per-sample relative abundances that sum to 100%. These TSS-normalized proportions were used for all composition summaries. For compositional visualization, taxa tables were log_2_-transformed and clustered in pheatmap using Euclidean distance and complete linkage; stacked barplots display the same proportions (top taxa selected by cohort-wide mean abundance). Alpha diversity (Shannon, Simpson, Inverse Simpson indexes) was computed at the species level from the proportion matrices; diversity is reported per site as median and interquartile range (IQR, 25–75th percentile) across samples, and per sample as individual values in the figures. Resistome prevalence for each antibiotic class was calculated as the number of positive samples (presence/absence) at cohort and site levels; per-sample ARG breadth was defined as the count of classes detected in each sample. Unless stated otherwise, all descriptive statistics were produced in R with data.table, ggplot2, pheatmap, and vegan. No formal hypothesis tests were performed in this study; the results are presented as descriptive and exploratory summaries to characterize patterns across sites.

## 5. Conclusions

This study revealed a shared bacterially dominated microbiome and a core resistome across three Sicilian WWTPs, with local variations reflecting environmental and anthropogenic influences. The widespread presence of β-lactam, macrolide, and aminoglycoside resistance genes confirms wastewater as a key hotspot for antimicrobial resistance dissemination. By providing region-specific metagenomic data from urban wastewater influents, this work contributes to the growing body of evidence supporting the integration of metagenomic monitoring into wastewater surveillance programs as crucial to support early detection, risk assessment, and One Health-oriented strategies that limit the environmental spread of antibiotic resistance.

## Figures and Tables

**Table 1:** Summary of sampling design, DNA pooling strategy, and number of sequenced composite samples per wastewater treatment plant (WWTP).

Parameter	Description
Sampling period	February 2022–December 2023
Sampling frequency	Weekly
Sampling location	Influent (before any treatment)
WWTPs included	Pantano D’Arci (Catania), Syracuse, Giarre (Catania), Sicily, Italy
Volume per weekly sample	100 mL
Initial processing	Individual concentration and DNA extraction for each weekly sample
Compositing level	DNA level (no influent-level compositing)
Pooling strategy	Equal pooling of DNA from 4 consecutive weekly samples
Composite sample frequency	Monthly
Composite samples per site	23
Total composite samples sequenced	69
Sequencing method	Shotgun metagenomic sequencing

## Data Availability

Data are available on reasonable request from the corresponding author.

## Supplementary Materials

The following supporting information can be downloaded at: https://www.mdpi.com/article/10.3390/antibiotics15020148/s1, Figure S1: Species rarefaction curves.

## Abbreviations

The following abbreviations are used in this manuscript:

AMR	Antimicrobial resistance
ARG	Antimicrobial resistance gene
HGT	Horizontal gene transfer
MGEs	Mobile genetic element
qPCR	Real-time quantitative PCR
WWTPs	Wastewater treatment plants
