# Supplementary material for: The Wastewater Resistome: A Shotgun Metagenomics Analysis of Urban Treatment Plants in Sicily
Source: Antibiotics (Basel). 2026 Feb 2;15(2):148. doi: 10.3390/antibiotics15020148 (PMC12937349; doi:10.3390/antibiotics15020148)
Supplement: Supplementary file 1 [file antibiotics-15-00148-s001.zip › antibiotics-4100810-supplementary.pdf]

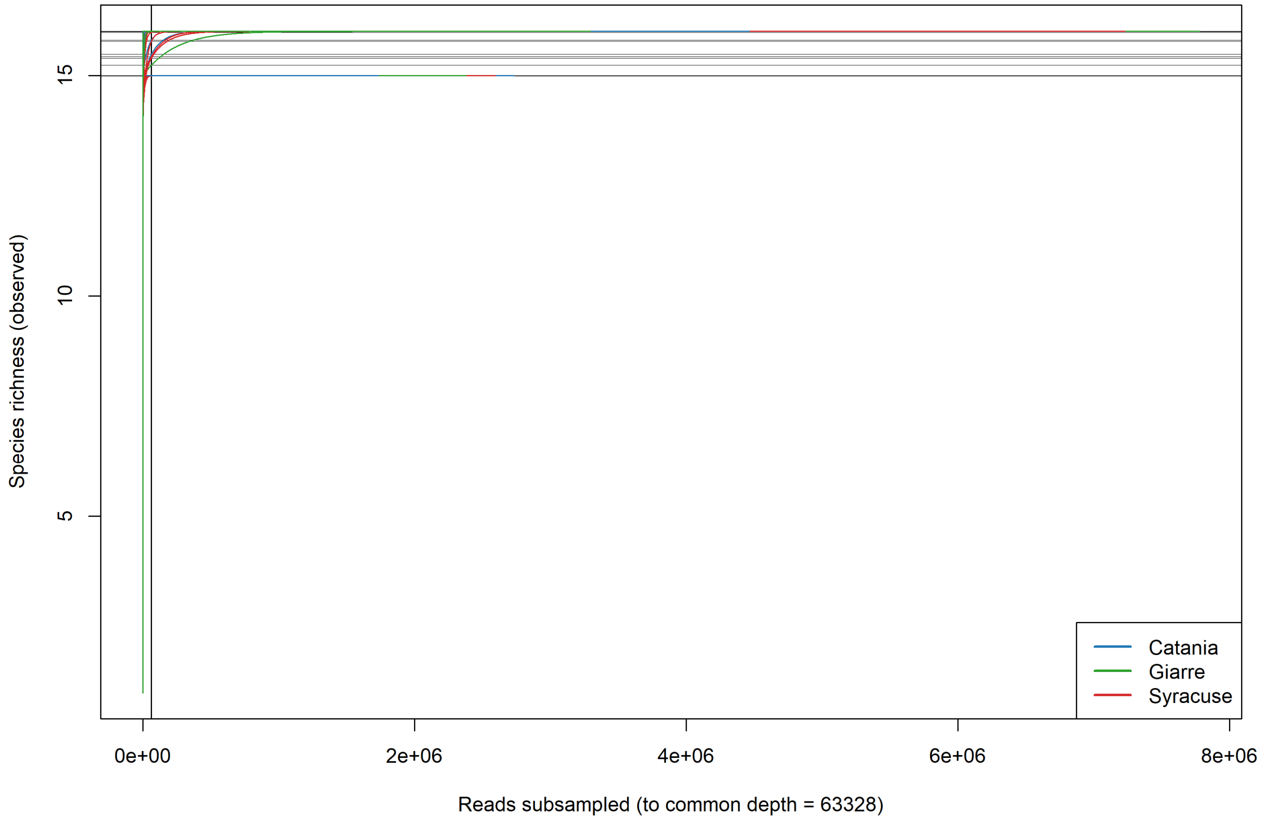

Figure S1. Species rarefaction curves. Rarefaction computed from the species abundance count table (post-decontamination, annotated/retained species). Curves were subsampled to the common minimum library size across samples and plateau rapidly, indicating adequate coverage for the retained species set.
